# Novel parathyroid hormone-based bone graft substitute, KUR-111, in treatment of tibial plateau fractures: a prospective, randomised, open-label, multicenter study

**DOI:** 10.1007/s00590-025-04645-2

**Published:** 2026-01-14

**Authors:** Nikolaos K Kanakaris, Michael J Raschke, Joe M Lane, James T Ryaby, Brent L Atkinson, Peter V Giannoudis

**Affiliations:** 1 Academic Department of Trauma and Orthopaedics, Leeds Teaching Hospitals NHS Trust, University of Leeds, Leeds, UK; 2 Klinik and Poliklinik fur Unfall- Hand- und Wiederherstellungschirurgie, University Hospital Münster, Münster, Germany; 3 Weill Cornell Medicine, Metabolic Bone Disease Service, Hospital for Special Surgery, New York, USA; 4 Kuros Biosciences BV, Schlieren, Switzerland; 5 Atkinson Biologics Consulting, Highlands Ranch, CO, USA; 6 NIHR Leeds Biomedical Research Centre, Chapel Allerton Hospital, Leeds, UK

**Keywords:** NATO treaty case, War injuries, Extremity trauma, Interdisciplinarity, Patient Distribution, DGU Trauma Network

## Abstract

**Background:**

The treatment of closed tibial plateau fractures (TPF) is complex and carries a risk of malunion. Parathyroid hormone (PTH) plays a key role in bone metabolism, and a PTH-peptide (PTH_1 − 34_) promotes bone healing. The objective was to evaluate the safety and efficacy of a novel PTH-based bone-graft-substitute (KUR-111) in the treatment of TPF.

**Methods:**

The study was a randomised, controlled, multicenter, open-label (dose-blinded), and dose-finding clinical trial. Subjects were randomised into 3 groups (iliac crest autograft (control); KUR-111-low; and high-dose TGplPTH1-34). The primary efficacy endpoint was the rate of union by computed tomography (CT) at 16weeks, as assessed by the Independent Radiologist Evaluation Panel (IREP).

**Results:**

A total of 183 TPF were enrolled and treated. The primary endpoint was met, as statistical non-inferiority was demonstrated for KUR-111-high compared with autograft at 16weeks. KUR-111-high significantly (*p* = 0.03) increased union rates compared to KUR-111-low (83.6%vs66.1%). IREP and a clinician-assessed composite score of fracture healing showed higher healing rates for KUR-111-high than KUR-111-low or autograft. Loss of reduction was minimal (0.4–0.9 mm) without significant differences (*p* > 0.10) among groups. Mean pain of the treated knee improved from baseline, with the least pain for KUR-111-high at all timepoints. Clinically significant donor-site pain was reported by 61.8% at discharge and remained in 12.2% of subjects at 104weeks. By 104weeks, analgesic use following KUR-111-high was less than one-half (9.8%vs24.1%), and opioid use was approximately 7-fold lower (1.6%vs12.1%) as compared to autograft.

**Conclusion:**

KUR-111-high has the potential to be a promising adjunctive therapy in the treatment of closed TPFs.

**Level of evidence:**

Therapeutic Level I.

## Introduction

Tibial plateau fractures (TPF) involve the articular surface of the proximal tibia and generally result from fragility or high-energy trauma. Treatment of TPF is complex, and there is a substantial risk of malunion, early arthritis, and deep infection [1–3]. Operative reduction and fixation of TPF addresses the post-traumatic knee instability and the significant articular displacement of intra-articular fragments.

In this procedure, the depressed articular surface is elevated and reduced anatomically, and the subchondral defect caused by compressed cancellous bone is commonly filled with bone graft or substitutes. According to a recent meta-analysis, autologous iliac crest bone graft remains the gold standard for treating TPF [4]. Complications associated with this second surgical site harvest include pain (acute and chronic), hematoma, infection and nerve injury [5–8]. To decrease these risks, synthetic bone graft substitutes (BGS) have been developed to spare the need for autograft harvest [9]. Although BGS are routinely utilised in clinical practice, there are only four randomised controlled trials (RCTs) [10–13], which evaluated their efficacy relative to autograft in treating TPFs. Of those four, only two [11, 13] reported union rates, neither utilised computed tomography (CT).

The KUR-111 BGS comprises a modified PTH-peptide (TGplPTH_1 − 34_) covalently bound to fibrin and mixed with hydroxyapatite/tricalcium phosphate (HA/TCP) granules. Previously, KUR-111 was investigated in a Phase-1 safety study of 10 patients with compression fractures of the distal radius. Nine out of 10 patients healed fractures by 12 weeks, and the remaining healed by 16 weeks. KUR-111 was generally well tolerated with no product-related serious adverse events (SAEs). A similar product, KUR-113, which contains the modified PTH-peptide and fibrin but lacks the HA/TCP granules, was investigated in a Phase-2 RCT versus standard care for treating open tibial shaft fractures [14]. The primary endpoint of fracture healing at 6 months was successfully met, which showed significantly higher prevalence of healing versus the control group (80.4% vs. 64.6%) [14]. 

The objective of the current study was to evaluate the safety and efficacy of KUR-111 in treating subjects with a closed TPF, using a randomised, open-label (dose-blinded), dose-finding design, compared to autograft, with fracture healing assessed by CT according to an independent radiographic panel.

## Materials and methods

### Study design

This was a Phase-2, prospective, randomised, controlled, open-label (dose-blinded), dose-finding, parallel-group, international multicenter study to evaluate the efficacy and safety of single implantation of an investigational product KUR-111 (2 doses), with internal fixation for the treatment of closed tibia plateau fractures (TPF) as compared with autologous bone grafting with internal fixation in an open procedure. All subjects provided written informed consent to participate. Regulatory and Independent Ethical Committee approval was obtained for each site before initiation; the study was conducted by the World Medical Association Declaration of Helsinki and was registered in ClinicalTrials.gov (NCT00409799).

The primary efficacy endpoint was the proportion of subjects with radiological fracture union by CT at 16 weeks, as assessed by the IREP using the criteria defined in Table 1. The secondary efficacy endpoints were (1) the loss of reduction of the tibial plateau / step-Off (in mm) by x-rays as compared to the immediate postoperative visit as measured by the Independent Radiographic Evaluation Panel (IREP); (2) the proportion of subjects with radiological fracture union by CT at 52 and 104 weeks assessed by IREP; (3) The number of subjects with secondary interventions (defined by surgical procedures for knee arthroplasty or fracture healing and non-invasive treatments to promote fracture healing) due to non-healing within 104 weeks; and (4) IREP and a clinical composite measure of fracture healing (level of pain upon weight bearing on fracture site, level of redness/swelling of pain upon weight bearing on fracture site and whether a secondary intervention was necessary to promote fracture healing).

**Table 1:** Radiographic criteria for the primary efficacy endpoint used by the independent radiographic evaluation panel (IREP)

Criteria	Description
Cortical bridging on at least one visible cortical plane, AND	Re-established cortical continuity of each of the cortical planes including the corresponding articular surface. Cortical bridging was assessed using the anteromedial, anterolateral, posteromedial and posterolateral sections.
Obliteration of fracture lines, AND	Scored as: 0: visible (lucent) fracture line with sharp fracture fragment margins. 1: partial filling in of fracture line (25%) *2: partial filling in of fracture line (50%) *3: extensive filling in of fracture line, but still visible (75%) *4: complete return of normal trabecular bone across fracture line (100%).
Dislocation of bone fragments compared to the postoperative film	A fragment that changed position (integration) and loses the interconnection to the surrounding structures, leading to a significant incongruence or step-off of the articular surfaces compared to the immediate postoperative film. Scoring: *0: no change compared to immediate postoperative *1: partial dislocation (still in contact to the surrounding structures with no significant change on the congruity or step-off of the articular surface) 2: complete dislocation (disintegration and significant change on the congruity or step-off of the articular surface) Fragment rotation (most displaced fragment in degrees

*Definition of endpoint criteria being met:

Cortical bridging: At least one of four cortices bridged was required to demonstrate bridging

Obliteration of fracture lines: A score of at least 2

Dislocation: 0 or 1 and no fragment rotation > 20 degrees

### Sample size

The sample size was determined based on healing rates of 85%-90% for the investigational and control treatments. If 56 subjects per group had evaluable results, the study had approximately 80% power to show non-inferiority of one of the doses relative to the autograft control, assuming a type-I error rate of 10%.

### Study population

A total of 208 subjects were enrolled and randomised in a 1:1:1 ratio across three groups at 30 participating sites in the European Union, Switzerland, and Australia from Jan 15, 2007, with the 104-week f/u completed June 7, 2011. Included subjects were at least 18 years of age, with radiological evidence of TPF that required grafting and fixation that occurred either alone or as part of a polytraumatic event (AO classification: 41B2, 41B3, 41C2, 41C3) and with a BMI of 16–33. The main exclusion criteria were patients with a high risk of amputation and open tibial plateau fractures, Gustilo-Anderson grade III.

After enrollment, chronic use (oral or parenteral treatment for more than five consecutive days) of COX2 inhibitors, steroids (except topical), and other PTH treatments was prohibited.

### Randomisation

Before surgery, subjects were assigned to one of the three treatment groups by a central randomisation procedure. The subject treatment assignment was stratified by AO classification. The randomisation was carried out in blocks of three in a ratio of 1:1:1 using a random number generator algorithm.

### Blinding

The study was deemed open-label due to the different technical procedures used for autograft and investigational treatments. To reduce bias, the investigators were blinded to the KUR-111 concentrations. The IREP assessed the primary and secondary endpoints and was blinded to the concentration of KUR-111. The patients, treating surgeons, and outcome assessors were not blinded to whether the patient underwent treatment with KUR-111 or the control.

### Study interventions

The fracture reduction, fixation, and grafting procedure was performed within 14 days of the TPF. Surgery was performed in accordance with the investigator’s standard of care, and only internal fixation (plates and/or screws) was used.

KUR-111 is comprised of fibrin sealant that is mixed with the peptide, TGplPTH_1 − 34_, and granules (comprising hydroxyapatite, HA, and tricalcium phosphate, TCP (Biomatlante, France)). TGplPTH_1 − 34_, was developed to allow slow-release of PTH_1 − 34_, during bone repair. The technology comprises a 12-amino acid linker, termed the TG-hook, that contains a transglutaminase substrate and a plasmin cleavage site. Upon mixing with a fibrin sealant, the TGplPTH_1 − 34_, peptide is covalently linked to fibrin by factor XIIIa as the fibrin clot forms. As the clot is gradually degraded in situ by plasmin, PTH_1 − 34_, is released locally [15, 16]. 

**Fig. 1:**
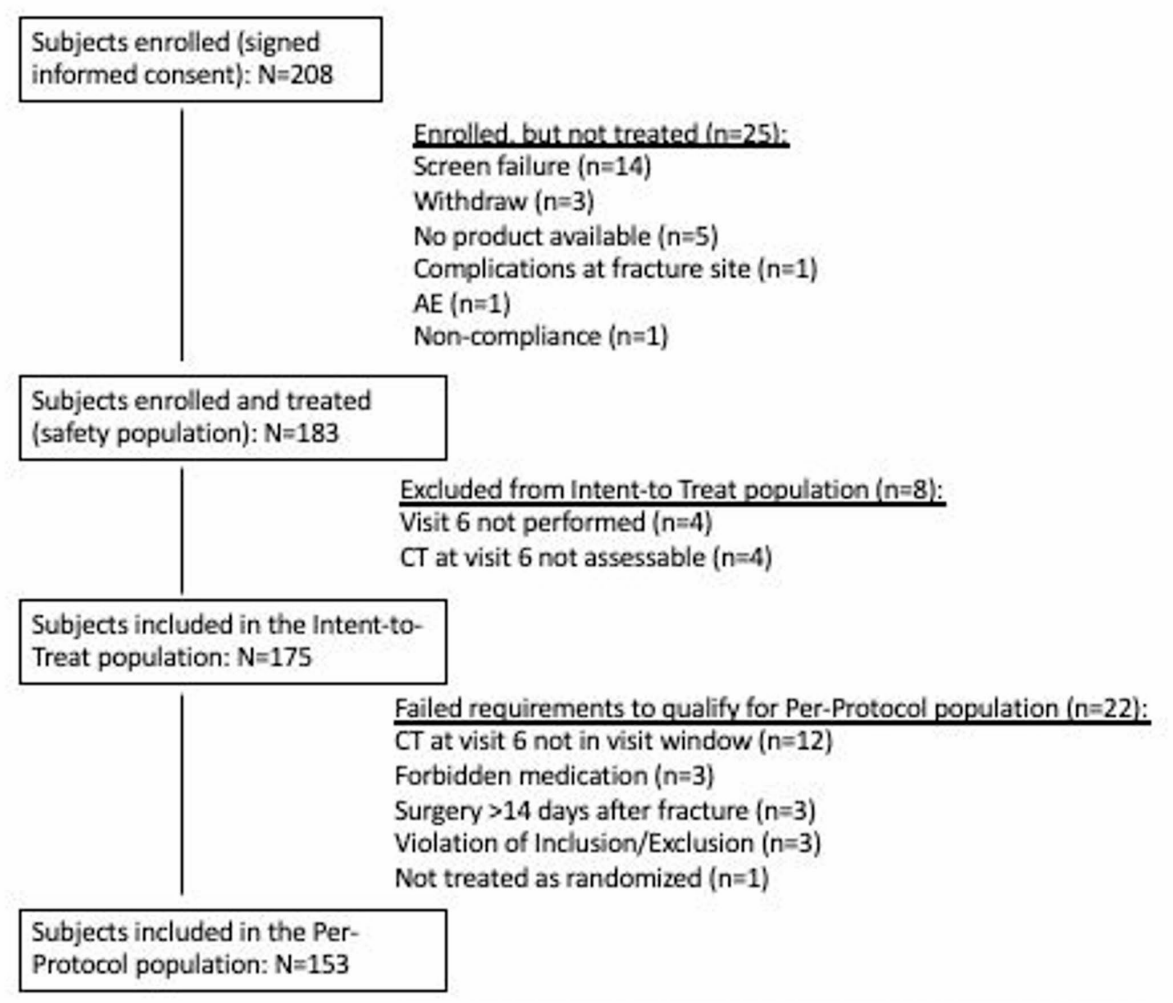
Consolidated Standards of Reporting Trials (CONSORT) flow diagram of the study

The KUR-111—low and—high contained 0.4 mg TGplPTH1-34 and 1.0 mg TGplPTH1-34/mL, respectively, in fibrin (Artiss, Baxter) and combined with HA/TCP granules, which creates a moldable, compression-resistant putty (Fig. 1). For the control, investigators were directed to harvest cancellous bone from the iliac crest. The graft volume was determined by the void volume to be filled.

### Radiographic assessments

X-rays were obtained at every follow-up visit. CT was performed at 16, 52 and 104 weeks, and the IREP performed all assessments. TPF Loss of reduction was determined by the distance between the surface of the most dislocated fragment and the longitudinal axis of the tibia using AP X-rays.

### Study outcomes

The radiograph reading methodology required two board-certified radiologists or orthopaedic surgeons to agree on the determination of fracture union. If consensus was not reached, a third board-certified evaluated the case.

### Statistical analysis

All safety analyses were based on the safety population (subjects treated), and efficacy analyses were based on the intention-to-treat (ITT) population (e.g., the safety population excluding those without a 16-week visit or evaluable CTs at 16 weeks). The primary statistical analysis aimed to demonstrate the non-inferiority of KUR-111 compared to autograft regarding radiographic fracture union at 16 weeks postoperatively. If the lower limit of the 90% two-sided confidence interval (CI) for the difference in union rates between subjects who received KUR-111 versus autograft was above − 15%, KUR-111 was considered at least as adequate as autograft. Fisher’s exact test assessed the difference in the primary endpoint between KUR-111 doses. The Wilcoxon rank-sum test detected statistical differences between groups for loss of reduction.

## Results

### Study population

A total of 208 subjects were enrolled. The Consolidated Standards of Reporting Trials (CONSORT) flow diagram shows that of the 183 subjects enrolled and treated (safety population), 175 were included in the Intent-to-Treat (ITT) population and 153 in the Per-Protocol (PP) population (Fig. 1). The safety population (treated) included 59 subjects treated with KUR-111-low, 63 with KUR-111-high and 61 with autograft.

**Fig. 2:**
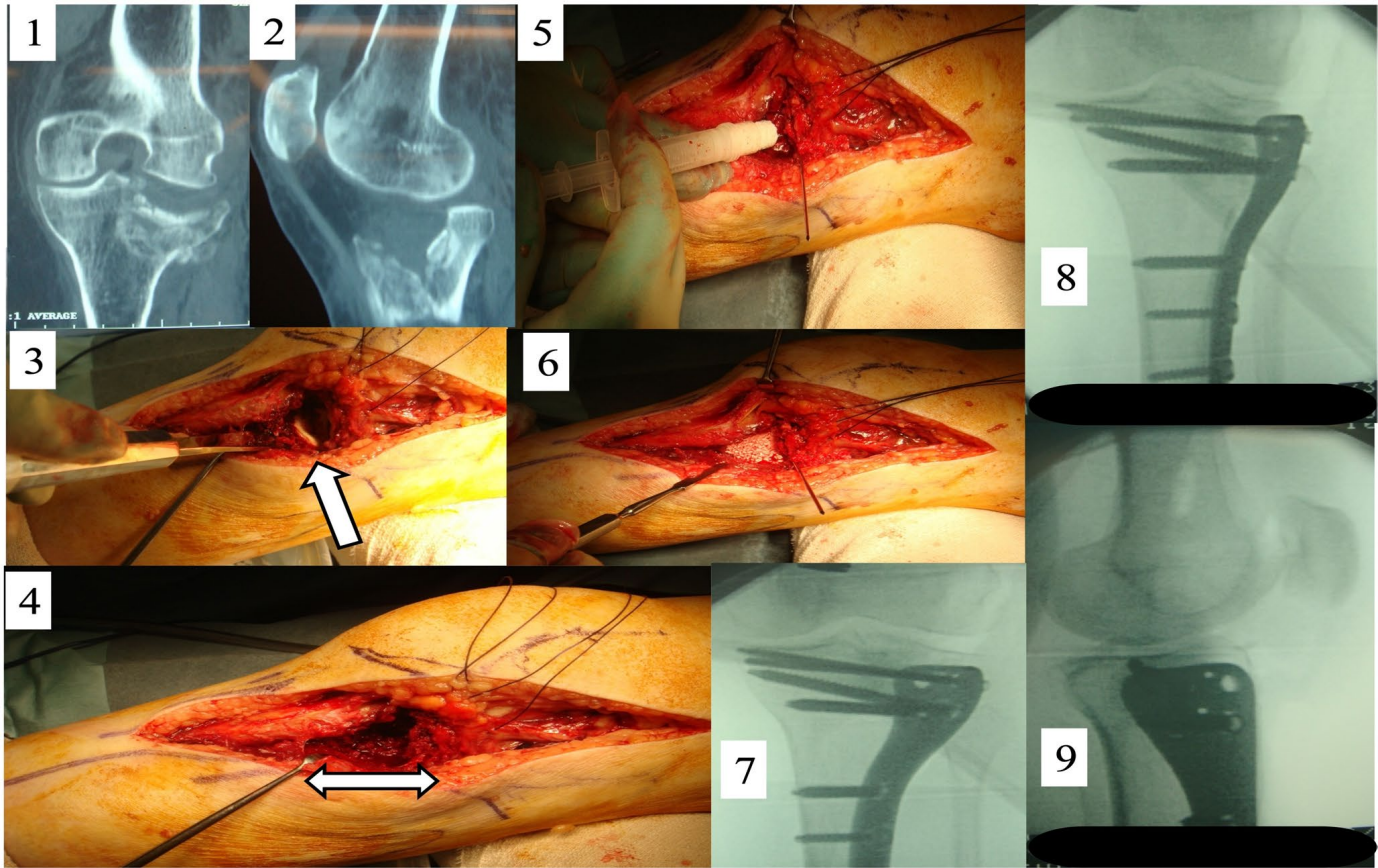
Surgical administration of KUR-111: 1. Axial CT image of a type II tibial plateau fractue in an elderly patient. 2. Sagittal CT image of a type II tibial plateau fractue in an elderly patient. 3. Intra-operative image following a lateral approach with arrow showing degree of depression of articular surface prior to reduction. 4. Intra-operative image with arrow showing degree of bone void created after reduction of articular surface. 5. Intra-opertaive image showing delivery of KUR-111 graft material to the bone void. 6. Intra-operative image showing filling of the void with the KUR-111 graft material. 7,8. AP intraoperative radiographic images showing the result of reconstruction. 9. Lateral intraoperative radiographic image showing the result of reconstruction

Subject demographics were similar across treatment groups (Table 2). The average age and BMI for the groups were approximately 50 years and 26 kg/m^2^, respectively. The proportion of current nicotine users was comparable among groups and ranged from 33.9% to 41.4%. Operative characteristics were also similar among the groups (Table 3). The most common fracture pattern was 41B3 (split compression fracture) (Fig. 2), which was found in 96 subjects (54.9%) and was comparably distributed among the three groups. There were no meaningful differences among groups for subjects with more severe 41C2 and 41C3 fractures. The surgery time, graft volume and duration of hospitalisation were also similar among groups.

**Table 2:** Demographic characteristics of the safety population

Characteristic	KUR-111-Low *n*/*N* (%)	KUR-111-High *n*/*N* (%)	Control Autograft *n*/*N* (%)
Male	36/59 (61.0)	37/63 (58.7)	34/61 (55.7)
Female	23/59 (39.0)	26/63 (41.3)	27/61 (44.3)
Age Mean +/- SD	49.7 +/- 14.9	48.2 +/- 15.2	53.2 +/- 15.9
BMI (kg/m^2^) Mean +/- SD	25.6 +/- 3.5	26.0 +/-3.8	26.3 +/- 3.8
Nicotine			
No	33/56 (58.9)	32/61 (52.5)	22/58 (37.9)
Former	4/56 (7.1)	7/61 (11.5)	13/58 (22.4)
Current	19/56 (33.9)	22/61 (39.7)	23/58 (41.4)

BMI, body mass index; N, total number; n, number of cases; NA, not applicable; SD, standard deviation

**Table 3:** Characteristics of fracture types, procedures and hospitalization of the ITT population

		KUR-111-Low *N* = 56 *n* (%)	KUR-111-High *N* = 61 *n* (%)	Autograft *N* = 58 *n* (%)	TOTAL *N* = 175 *n* (%)
*Type of fracture per AO/OTA Classification*					
41B2	Depression fracture	9 (16.1)	16 (26.2)	10 (17.2)	35 (20.0)
41B3	Split depression fracture	33 (58.9)	29 (47.5)	34 (58.6)	96 (54.9)
41C2	Simple articular, wedge or multi-fragmentary metaphyseal fracture	9 (16.1)	3 (4.9)	3 (5.2)	15 (8.6)
41C3	Fragmentary or multi-fragmentary metaphyseal fracture	5 (8.9)	13 (21.3)	11 (19.0)	29 (16.6)
Surgery time mean (SD), hours		1.8 (1.1)	1.6 (0.9)	1.6 (0.9)	NA
Graft volume mean (SD), cc		5.1 (1.9)	5.3 (1.8)	5.2 (2.2)	NA
Duration of hospitalization mean (SD), days		14.5 (9.7)	13.3 (6.2)	15.0 (6.4)	NA

cc, cubic centimeters; ITT, intention to treat; N, total number; n, number of cases; NA, not applicable; SD, standard deviation

### Efficacy assessments

**Fig. 3:**
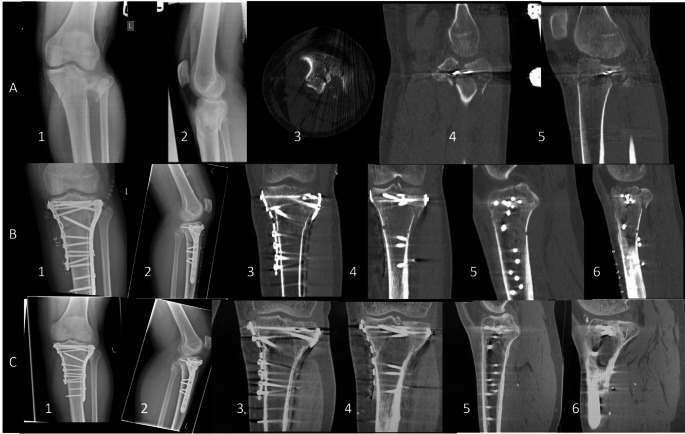
Male 27yoa, Schatzker VI or AO/ASIF 41C3 fracture with large lateral condyle joint depression (visible at A1 plain x-ray), managed initially with a bridging the knee external fixator and 2 percutaneous buried 1.6 mm k-wires (visible at A4 and A5 cuts) as means of temporary reduction/fixation till definite fixation. A dual surgical approach and plate fixation with joint surface rafting with screws and augmentation with the autologous bone grafting (anterior iliac crest) was performed 6 days later. Row A includes plain x-rays and CT scan slices on arrival: AP X-ray (A1); lateral x-ray (A2); axial cut at joint level (A3); coronal cut (A4); sagittal cut (A5). Row B includes plain x-rays and CT scan slices immediately postoperatively: AP x-ray (B1); lateral x-ray (B2); coronal cuts (B3 & 4); sagittal cuts (B5 & 6). Row C includes plain x-rays and CT scan slices at 12 months postoperatively: AP x-ray (C1); lateral x-ray (C2); coronal cuts (C3 & 4); sagittal cuts (C5 & 6). In comparison to immediate postop imaging there is evidence of complete healing and absence of secondary collapse at the comminuted lateral tibial plateau joint surface

**Fig. 4:**
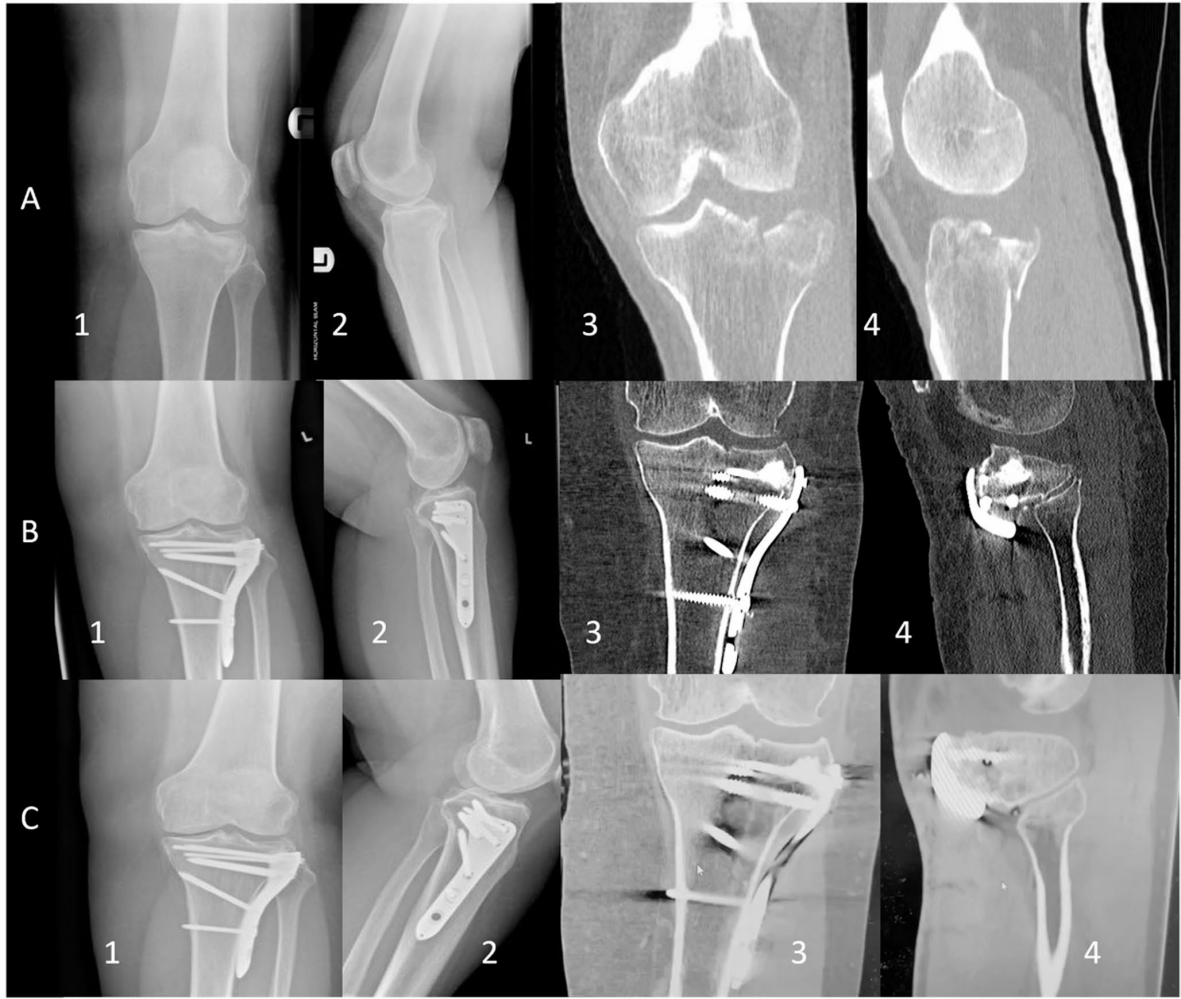
Female 54yoa, Schatzker III or AO/ASIF 41B2 fracture with comminution and depression of the lateral condyle joint surface was managed with an anterolateral surgical approach and plate fixation with joint surface rafting with screws and augmentation with the KUR-111 high bone graft substitute (clearly visible at B3 and B4 CT scan slices). Row A includes plain x-rays and CT scan slices on arrival: AP x-ray (A1); lateral x-ray (A2); coronal cut (A4); sagittal cut (A5). Row B includes plain x-rays and CT scan slices immediately postoperatively: AP x-ray (A1); lateral x-ray (A2); coronal cut (A4); sagittal cut (A5). Row C includes plain x-rays and CT scan slices at 12 months postoperatively: AP x-ray (A1); lateral x-ray (A2); coronal cut (A4); sagittal cut (A5). In comparison to immediate postop imaging, there is evidence of complete healing and the absence of secondary collapse at the comminuted lateral tibial plateau joint surface

The proportion of subjects with fracture healing at 16 weeks (primary efficacy endpoint) was significantly more significant [*p* = 0.03] in the KUR-111-high group (51 of 61; 83.6%) as compared to the KUR-111-low group (37 of 56; 66.1%) and was without statistical significance as compared to the autograft group (50 of 59, 84.7%) (Table 4). CT and X-ray imaging of two patients treated with autograft and KUR-111-high are shown in Figs. 3 and 4, respectively. The primary endpoint was met because statistical non-inferiority was demonstrated for KUR-111-high compared to autograft at 16 weeks for the ITT population. Non-inferiority was also shown for the PP population. At 52 and 104 weeks, the proportion of subjects with radiological union increased over 16 weeks and ranged from 96.2 to 100% across all groups.

**Table 4:** Radiological fracture union based on CT scan and assessed by the IREP over time for the ITT population

Time (weeks)	KUR-111-Low *n*/*N* (%) [90% CI*]	KUR-111-High *n*/*N* (%) [90% CI*]	Autograft *n*/*N* (%) [90% CI*]	90% CI in the comparative analysis between	
				KUR-111-low vs. Autograft	KUR-111-high vs. Autograft
16**	37/56 (66.1) [54.3–76.5]	51/61 (83.6)† [73.8–90.8]	50/59 (84.7) [74.9–91.8]	-0.323 to -0.045	-0.125 to 0.104‡
52	50/52 (96.2) [88.4–99.3]	58/58 (100) [95.0-100]	54/55 (98.2) [91.7–99.9]	-0.095 to 0.045‡	-0.030 to 0.083‡
104	42/43 (97.7) [89.4–99.9]	56/56 (100) [94.8–100]	46/47 (97.9) [90.3–99.9]	-0.081 to 0.074‡	-0.029 to 0.097‡

* Clopper-Pearson exact Confidence Interval (CI)

** primary endpoint

†= *P* < 0.05 versus KUR-111-low using Fisher’s exact test

‡ non-inferior to autograft since <-0.15

Non-inferiority for the primary endpoint was met for KUR-111-high for the PP population also

CT: computerised tomography; N: total number; n: number of cases; NA: not applicable; IREP: Independent Radiographic Evaluation Panel

A composite healing analysis included both the CT-based method from IREP (Table 1) and clinical healing as assessed by the Investigator. At 16 weeks, KUR-111-high had the highest composite incidence of healed subjects (72.1%), followed by autograft (63.8%) and KUR-111-low (57.1%). KUR-111-high also had the highest composite incidence of subjects healed at 52 and 104 weeks (Figs. 3 and 4; Table 5).

**Table 5:** Fracture healing as assessed by combining CT scan findings (evaluated by the IREP) and the clinical assessment of the investigator † for the ITT cohort

Time (weeks)	KUR-111-Low *n*/*N* (%) [90% CI*]	KUR-111-High *n*/*N* (%) [90% CI*]	Autograft *n*/*N* (%) [90% CI*]
16	32/56 (57.1) [45.3–68.4]	44/61 (72.1) [61.2–81.4]	37/59 (62.7) [51.2–73.2]
52	46/50 (92.0) [82.6–97.2]	55/58 (94.8) [87.2–98.6]	50/55 (90.9) [81.8–96.3]
104	39/42 (92.9) [82.6–98.0]	54/56 (96.4) [89.2–99.4]	44/47 (93.6) [84.3–98.2]

*Clopper-Pearson exact Confidence Interval (CI)

†= clinically healed if the level of redness, swelling, or pain upon weight bearing on the fracture site was “no/ minimal” and no secondary intervention was required to promote fracture healing

CT scan: computerized tomography scan; IREP: Independent Radiographic Evaluation Panel; ITT: intention to treat cohort; N: overall number; n: number of cases

The mean loss of reduction (step-off) was small, without statistical significance and ranged from 0.4 to 0.9 mm among all groups and from 16 to 104 weeks (Table 6).

**Table 6:** Loss of reduction (Step-Off) based on x-ray as compared to the immediate post-operative visit as assessed by the IREP for the ITT population

Time (weeks)	KUR-111-Low Mean (SD) mm	*P*-value† low vs. autograft	KUR-111-High Mean (SD) mm	*P*-value† high vs. autograft	Autograft Mean (SD) mm
16	0.4 (0.4)	0.21	0.6 (0.5)	0.81	0.6 (0.5)
52	0.6 (0.5)	0.29	0.7 (0.5)	0.10	0.6 (0.6)
104	0.8 (0.9)	0.89	0.9 (0.8)	0.14	0.7 (0.7)

†= Wilcoxon rank sum test

IREP, Independent Radiographic Evaluation Panel; ITT, intention to treat population; N, total number; n, number of cases; NA, not applicable; SD, standard deviation

A categorical analysis showed minimal (< 2 mm category) loss of reduction (step-off) at 16, 52 and 104 weeks for all groups in the majority of the subjects (Table 7). By 104 weeks, the incidence of less than 2 mm loss of reduction was 88.6%, 87.8% and 95.2% in the KUR-111-low, KUR-111-high and autograft groups, respectively. At all timepoints, no subjects experienced a loss of reduction of greater than 5 mm.

**Table 7:** Categorized loss of Reduction- Step-Off based on x-ray as compared to the immediate post-operative visit as assessed by the IREP for the ITT population at 104 weeks

Loss of Reduction (mm)	KUR-111-Low *n*/*N* (%)	KUR-111-High *n*/*N* (%)	Autograft *n*/*N* (%)
16 weeks			
< 2	49/50 (98.0)	56/56 (100)	50/50 (100)
2–5	1/50 (2.00)	0/56 (0)	0/50 (0)
> 5	0/50 (0)	0/56 (0)	0/50 (0)
52 weeks			
< 2	42/43 (97.7)	52/52 (100)	48/49 (98.0)
2–5	1/43 (2.3)	0/52 (0)	1/49 (2.0)
> 5	0/43 (0)	0/52 (0)	0/49 (0)
104 weeks			
< 2	31/35 (88.6)	43/49 (87.8)	40/42 (95.2)
2–5	4/35 (11.4)	6/49 (12.2)	2/42 (4.8)
> 5	0/35 (0)	0/49 (0)	0/42 (0)

IREP, Independent Radiographic Evaluation Panel; ITT, intention to treat population; mm, millimetre; N, total number; n, number of cases

At baseline, VAS pain of the treated knee was similar among all three groups. VAS knee pain scores improved from baseline for each group (Table 8). At each timepoint, the least VAS pain was for subjects treated with KUR-111-high.

**Table 8:** VAS Pain* of the treated knee for the ITT population

Time (weeks)	KUR-111-Low Mean (SD) [range]	KUR-111-High Mean (SD) [range]	Autograft Mean (SD) [range]
Baseline	32.3 (32.8) [0–95]	36.5 (32.6) [0-101]	36.6 (34.3) [0–99]
16	18.1 (23.4) [0–89]	10.5 (13.9) [0–60]	19.4 (22.7) [0–82]
52	17.2 (24.0) [0–79]	13.8 (19.3) [0–71]	14.7 (21.0) [0–87]
104	12.6 (20.0) [0–67]	11.0 (19.3) [0–75]	12.7 (17.7) [0–94]

*VAS was defined as pain in the affected knee in the past week

ITT: intention to treat population; SD: standard deviation; VAS: visual analogue scale

### Safety evaluations

There were four deaths reported, and none of these were related to the treatment or procedure. These deaths were attributed to multi-organ failure in a subject with 14 SAEs, inhalatory pneumopathy post alcoholic coma, bilateral chronic subdural hematoma and prostate carcinoma. Five SAEs were reported, and none were related to the treatment or procedure. Three (5.4%) were for subjects treated with KUR-111-low, and two for autograft (3.4%). The proportion of subjects with non-serious AEs was comparable among groups, 22.4% for KUR-111 treatment versus 27.6% for autograft.

Within 104 weeks after surgery for the ITT group, the number of subjects who had secondary interventions was low for all groups and ranged from 0 to 5.4%. Subjects treated with KUR-111- high had no secondary interventions (0 of 61, 0%). Additional details, including the reason for secondary interventions, are described in Table 9.

**Table 9:** Secondary interventions within 104 weeks for the ITT group

	KUR-111-Low *n*/*N* (%) [90% CI]	KUR-111-High *n*/*N* (%) [90% CI]	Autograft *n*/*N* (%) [90% CI]
**Incidence**	3/56 (5.4)† [1.5–13.3%]	0/61 (0.0) [0.0-4.8%]	1/59 (1.7) ‡ [0.1–7.8%]

† Reason: Two surgical procedures to promote fracture healing and one knee arthroplasty

‡ Reason: One surgical procedure to promote fracture healing

CI: confidence interval; ITT: intention to treat population; N: total number; n: number of cases

At discharge, 61.8% of autograft subjects reported clinically significant donor site pain of 20 + mm [17, 18], with a mean and maximum VAS of 33 mm and 95 mm, respectively (Table 10). Over time, mean pain and the incidence of subjects that reported pain decreased, although, by 104 weeks, 12.2% of subjects continued to report clinically significant graft harvest site pain.

**Table 10:** Donor site pain* as assessed with the VAS for the autograft group

Time (weeks)	VAS mm Mean (SD)	Subjects with VAS > 20 mm *n*/*N* (%)	VAS mm Range
Discharge	33.0 (24.8)	34/55 (61.8)	0–95
16	6.5 (13.4)	5/48 (10.4)	0–81
52	5.2 (8.0)	5/47 (10.6)	0–32
104	6.3 (12.9)	5/41 (12.2)	0–71

*Defined as a hip donor site in the past week

N: total number; n: number of cases; ITT: intention to treat population; mm: millimetre; SD: standard deviation; VAS: visual analogue scale

At 16 weeks, all subjects from all groups reported analgesic use, which decreased substantially over time for all three groups. By 52 and 104 weeks, subjects treated with autograft reported the highest percentage of analgesic use, and those treated with KUR-111-high reported the least. By 104 weeks, analgesic use for the KUR-111-high group was less than one-half of that reported for autograft (9.8% vs. 24.1%) (Table 11). The opioid component of analgesic use demonstrated greater than 7-fold lower use with KUR-111-high as compared to autograft by 104 weeks (1.6% vs. 12.1%).

**Table 11:** Use of analgesics at different time points

Duration (weeks)	Category or Subcategory	KUR-111-Low *n* (%) *N* = 56	KUR-111-High *n* (%) *N* = 61	Autograft *n* (%) *N* = 58
< 16	All analgesic	56 (100)	61 (100)	58 (100)
	Opioid	18 (32.1)	11 (18.0)	11 (9.0)
16–52	All analgesic	11 (19.6)	7 (11.5)	12 (20.7)
	Opioid	5 (8.9)	4 (6.6)	9 (15.5)
53–104	All analgesic	8 (14.3)	6 (9.8)	14 (24.1)
	Opioid	2 (3.6)	1 (1.6)	7 (12.1)

N: total number; n: number of cases

Additional safety assessments performed by the IREP revealed no evidence of ectopic bone formation at the fracture site at week 104 for both of the KUR-111 treatments (0 of 99; 0%) and one for autograft treatment (1 of 43; 2.3%).

## Discussion

The primary study endpoint was met because statistical non-inferiority for fracture unions was demonstrated for KUR-111-high compared to autograft at 16 weeks. KUR-111-high treatment improved radiological and clinical fracture healing, patient-reported VAS pain scores, and analgesic use compared with KUR-111-low and autograft treatments. Relative to KUR-111-low at 16 weeks, subjects treated with KUR-111-high showed significantly higher fracture union rates, as assessed by independent radiological evaluations. The composite score for fracture healing combined standard clinical assessments with radiological assessments to incorporate a clinical perspective on healing. The resulting composite score showed higher healing rates for KUR-111-high than KUR-111-low or autograft at 16, 52, and 104 weeks. Mean VAS pain of the treated knee improved from baseline for all treatment groups, with the least pain reported for KUR-111-high at all time points. Correspondingly, analgesic use for KUR-111-high was less than one-half, and opioid use was approximately 7-fold lower than that of autograft-treated subjects. There were no safety concerns following the use of KUR-111.

The PP population was evaluated to determine whether the exclusion of ITT subjects with additional missing radiographic data or other protocol violations may have biased the primary efficacy endpoint. Similar to the ITT evaluation, statistical non-inferiority for KUR-111-high was met, and thus, there was no bias due to missing radiographs.

Similar to numerous reports in the literature [6–8, 19, 20], autograft harvest resulted in a high percentage of subjects with acute, perioperative pain in the iliac crest harvest site and clinically significant pain in 12.2% of subjects at 2 years. The autograft harvest site also required a drain, limiting patient mobility during the perioperative period. No difference in mean operation time was reported to the autograft group, which was comparable to other reports [11, 13]. 

A different osteoinductive molecule, bone morphogenetic protein-2 (BMP-2), has been evaluated for the treatment of complex TPFs. Heterotopic bone formation occurred at a ten-fold higher rate- in 59% of subjects compared to 5.9% without BMP-2 treatment [21]. The publication also reported increased reoperation rate for BMP-2 treated patients [21]. In contrast, the current study demonstrated no heterotopic bone formation and no re-operations following KUR-111-high treatment.

The strengths of this study included the randomised, multicenter and controlled with the gold standard autograft design, inclusion of a range of fracture types including the more severe AO 41C2 and 41C3 fractures, evaluation of patient-reported outcomes (VAS), CT evaluated by an independent radiographic panel, a clinical assessment of fracture healing, and XR for assessment of subsidence/step off at both short-term (16 weeks) and mid-term (2 years) follow-up.

Limitations were that since TPF-associated injuries to ligaments, nerve and menisci were not assessed, there is a possibility that concomitant ligamentous injury may have contributed to the pain outcomes. Additionally, although the minimal drop-off observed would be expected to slow the progression of osteoarthritis, there was no direct measure of osteoarthritis, and additional longer-term studies would be required.

## Conclusions

Administration of KUR-111 as adjunctive therapy in patients with a closed TPF may provide comparable fracture healing without the pain and morbidity associated with autograft harvest.

## Data Availability

No datasets were generated or analysed during the current study.

## References

[1] Bormann M, Neidlein C, Neidlein N, Ehrl D, Jorgens M, Berthold DP, Bocker W, Holzapfel BM, Furmetz J (2023) High prevalence of persistent measurable postoperative knee joint laxity in patients with tibial plateau fractures treated by open reduction and internal fixation (ORIF). J Clin Med 12(17). 10.3390/jcm12175580 10.3390/jcm12175580 PMC10488731 37685647

[2] Hartwich M, Lans J, Jupiter JB, Babst R, Regazzoni P, Dell’Oca AF (2023) Joint depression in tibial plateau fractures: to bone graft or not to bone graft? Injury. 10.1016/j.injury.2023.02.050 10.1016/j.injury.2023.02.050 36894468

[3] Kantor AH, Clapp I, O’Neill DC, Daryoush JR, Myhre LA, Marchand L, Haller JM (2023) Tibial plateau fractures complicated by compartment syndrome are associated with a 3 times higher cost of care. J Orthop Trauma 37(11):568–573. 10.1097/BOT.0000000000002674 37459502 10.1097/BOT.0000000000002674

[4] Azi ML, Aprato A, Santi I, Kfuri M Jr., Masse A, Joeris A (2016) Autologous bone graft in the treatment of post-traumatic bone defects: a systematic review and meta-analysis. BMC Musculoskelet Disord 17(1):465. 10.1186/s12891-016-1312-4 27829447 10.1186/s12891-016-1312-4 PMC5103502

[5] Dimitriou R, Mataliotakis GI, Angoules AG, Kanakaris NK, Giannoudis PV (2011) Complications following autologous bone graft harvesting from the Iliac crest and using the RIA: a systematic review. Injury 42 Suppl 2S3–15. 10.1016/j.injury.2011.06.015 10.1016/j.injury.2011.06.015 21704997

[6] Heneghan HM, McCabe JP (2009) Use of autologous bone graft in anterior cervical decompression: morbidity & quality of life analysis. BMC Musculoskelet Disord 10:158. 10.1186/1471-2474-10-158 20015365 10.1186/1471-2474-10-158 PMC2806333

[7] Myeroff C, Archdeacon M (2011) Autogenous bone graft: donor sites and techniques. J Bone Joint Surg Am 93(23):2227–2236. 10.2106/JBJS.J.01513 22159859 10.2106/JBJS.J.01513

[8] Silber JS, Anderson DG, Daffner SD, Brislin BT, Leland JM, Hilibrand AS, Vaccaro AR, Albert TJ (2003) Donor site morbidity after anterior Iliac crest bone harvest for single-level anterior cervical discectomy and fusion. Spine (Phila Pa 1976) 28(2):134–139. 10.1097/00007632-200301150-00008 12544929 10.1097/00007632-200301150-00008

[9] Goff T, Kanakaris NK, Giannoudis PV (2013) Use of bone graft substitutes in the management of tibial plateau fractures. Injury 44 Suppl 1S86–94. 10.1016/S0020-1383(13)70019-6 10.1016/S0020-1383(13)70019-6 23351879

[10] Heikkila JT, Kukkonen J, Aho AJ, Moisander S, Kyyronen T, Mattila K (2011) Bioactive glass granules: a suitable bone substitute material in the operative treatment of depressed lateral tibial plateau fractures: a prospective, randomized 1 year follow-up study. J Mater Sci Mater Med 22(4):1073–1080. 10.1007/s10856-011-4272-0 21431354 10.1007/s10856-011-4272-0

[11] Hofmann A, Gorbulev S, Guehring T, Schulz AP, Schupfner R, Raschke M, Huber-Wagner S, Rommens PM, Group CES (2020) Autologous Iliac bone graft compared with biphasic hydroxyapatite and calcium sulfate cement for the treatment of bone defects in tibial plateau fractures: A Prospective, Randomized, Open-Label, multicenter study. J Bone Joint Surg Am 102(3):179–193. 10.2106/JBJS.19.00680 31809394 10.2106/JBJS.19.00680 PMC7508276

[12] Pernaa K, Koski I, Mattila K, Gullichsen E, Heikkila J, Aho A, Lindfors N (2011) Bioactive glass S53P4 and autograft bone in treatment of depressed tibial plateau fractures - a prospective randomized 11-year follow-up. J long Term Eff Med Implants 21(2):139–148. 10.1615/jlongtermeffmedimplants.v21.i2.40 22043972 10.1615/jlongtermeffmedimplants.v21.i2.40

[13] Russell TA, Leighton RK, Alpha BSMTPFSG (2008) Comparison of autogenous bone graft and endothermic calcium phosphate cement for defect augmentation in tibial plateau fractures. A multicenter, prospective, randomized study. J Bone Joint Surg Am 90(10):2057–2061. 10.2106/JBJS.G.01191 18829901 10.2106/JBJS.G.01191

[14] Orbeanu V, Haragus H, Crisan D, Cirstoiu C, Ristic B, Jamieson V (2022) Novel parathyroid Hormone-Based bone Graft, KUR-113, in treatment of acute open tibial shaft fracture: A Phase-2 randomized controlled trial. J Bone Joint Surg Am 104(5):441–450. 10.2106/JBJS.20.02109 34971551 10.2106/JBJS.20.02109

[15] Arrighi I, Mark S, Alvisi M, von Rechenberg B, Hubbell JA, Schense JC (2009) Bone healing induced by local delivery of an engineered parathyroid hormone prodrug. Biomaterials 30(9):1763–1771. 10.1016/j.biomaterials.2008.12.023 19124152 10.1016/j.biomaterials.2008.12.023

[16] Schense JC, Hubbell JA (1999) Cross-linking exogenous bifunctional peptides into fibrin gels with factor XIIIa. Bioconjug Chem 10(1):75–81. 10.1021/bc9800769 9893967 10.1021/bc9800769

[17] Baumhauer J, Pinzur MS, Donahue R, Beasley W, DiGiovanni C (2014) Site selection and pain outcome after autologous bone graft harvest. Foot Ankle Int 35(2):104–107. 10.1177/1071100713511434 24227683 10.1177/1071100713511434

[18] Baumhauer JF, Glazebrook M, Younger A, Quiton JD, Fitch DA, Daniels TR, DiGiovanni CW (2020) Long-term autograft harvest site pain after ankle and hindfoot arthrodesis. Foot Ankle Int 41(8):911–915. 10.1177/1071100720920846 32432488 10.1177/1071100720920846

[19] Arrington ED, Smith WJ, Chambers HG, Bucknell AL, Davino NA (1996) Complications of Iliac crest bone graft harvesting. Clin Orthop Relat Res 329300–309. 10.1097/00003086-199608000-00037 10.1097/00003086-199608000-00037 8769465

[20] Goulet JA, Senunas LE, DeSilva GL, Greenfield ML (1997) Autogenous Iliac crest bone graft. Complications and functional assessment. Clin Orthop Relat Res 33976–81. 10.1097/00003086-199706000-00011 10.1097/00003086-199706000-00011 9186204

[21] Boraiah S, Paul O, Hawkes D, Wickham M, Lorich DG (2009) Complications of Recombinant human BMP-2 for treating complex tibial plateau fractures: a preliminary report. Clin Orthop Relat Res 467(12):3257–3262. 10.1007/s11999-009-1039-8 19693635 10.1007/s11999-009-1039-8 PMC2772910

